# A Cell-surface Phylome for African Trypanosomes

**DOI:** 10.1371/journal.pntd.0002121

**Published:** 2013-03-21

**Authors:** Andrew P. Jackson, Harriet C. Allison, J. David Barry, Mark C. Field, Christiane Hertz-Fowler, Matthew Berriman

**Affiliations:** 1 Pathogen Genomics Group, Wellcome Trust Sanger Institute, Wellcome Trust Genome Campus, Hinxton, Cambridge, England, United Kingdom; 2 Department of Infection Biology, Institute of Infection and Global Health, University of Liverpool, Liverpool, England, United Kingdom; 3 Department of Pathology, University of Cambridge, Cambridge, England, United Kingdom; 4 Institute of Infection, Immunity and Inflammation, University of Glasgow, Glasgow, Scotland, United Kingdom; 5 Centre for Genomic Research, Institute of Integrative Biology, Biosciences Building, University of Liverpool, Liverpool, England, United Kingdom; Yale School of Public Health, United States of America

## Abstract

The cell surface of *Trypanosoma brucei*, like many protistan blood parasites, is crucial for mediating host-parasite interactions and is instrumental to the initiation, maintenance and severity of infection. Previous comparisons with the related trypanosomatid parasites *T. cruzi* and *Leishmania major* suggest that the cell-surface proteome of *T. brucei* is largely taxon-specific. Here we compare genes predicted to encode cell surface proteins of *T. brucei* with those from two related African trypanosomes, *T. congolense* and *T. vivax*. We created a cell surface phylome (CSP) by estimating phylogenies for 79 gene families with putative surface functions to understand the more recent evolution of African trypanosome surface architecture. Our findings demonstrate that the transferrin receptor genes essential for bloodstream survival in *T. brucei* are conserved in *T. congolense* but absent from *T. vivax* and include an expanded gene family of insect stage-specific surface glycoproteins that includes many currently uncharacterized genes. We also identify species-specific features and innovations and confirm that these include most expression site-associated genes (*ESAGs*) in *T. brucei*, which are absent from *T. congolense* and *T. vivax*. The CSP presents the first global picture of the origins and dynamics of cell surface architecture in African trypanosomes, representing the principal differences in genomic repertoire between African trypanosome species and provides a basis from which to explore the developmental and pathological differences in surface architectures. All data can be accessed at: http://www.genedb.org/Page/trypanosoma_surface_phylome.

## Author Summary

The African trypanosome (*Trypanosoma brucei*) is a single-celled, vector-borne parasite that causes Human African Trypanosomiasis (or ‘sleeping sickness’) throughout sub-Saharan Africa and, along with related species *T. congolense* and *T. vivax*, a similar disease in wild and domestic animals. Together, the African trypanosomes have significant effects on human and animal health and associated costs for socio-economic development in Africa. Genes expressed on the trypanosome cell surface are instrumental in causing disease and sustaining infection by resisting the host immune system. Here we compare repertoires of genes with predicted cell-surface expression in *T. brucei*, *T. congolense* and *T. vivax* and estimate the phylogeny of each predicted cell-surface gene family. This ‘cell-surface phylome’ (CSP) provides a detailed analysis of species-specific gene families and of gene gain and loss in shared families, aiding the identification of surface proteins that may mediate specific aspects of pathogenesis and disease progression. Overall, the CSP suggests that each trypanosome species has modified its surface proteome uniquely, indicating that *T. brucei*, *T. congolense* and *T. vivax* have subtly distinct mechanisms for interacting with both vertebrate and insect hosts.

## Introduction

African trypanosomes (*Trypanosoma* spp. section Salivaria) are unicellular hemoparasites of vertebrates. They are transmitted by Tsetse flies (*Glossina* spp.) and cause endemic disease throughout sub-Saharan Africa. African trypanosomes include *T. brucei* which causes Human African Trypanosomiasis (‘sleeping sickness’) and, along with two related species *T. congolense* and *T. vivax*, a similar disease in domestic and wild animals (‘nagana’). Although the incidence of human disease has recently declined [1], there remains an estimated 30,000 cases per year [2]; while total losses in agricultural productivity due to animal disease across Tsetse-infested Africa are estimated to be US$4.75 billion per annum [3]. The combined effects of African trypanosomes on humans and livestock are a significant threat to public and veterinary health, and wider socio-economic development [4].

The first genomic comparisons between *T. brucei* and related trypansomatid parasites, *T. cruzi* and *Leishmania major*, which cause Chagas disease and leishmaniasis in humans respectively, showed that most genes are widespread and arranged into regions of conserved synteny [5]–[7]. By contrast, it was also apparent that the gene families likely encoding cell surfaces molecules were non-homologous and largely lineage-specific [8]–[9]. In the vertebrate host, the *T. brucei* surface is dominated by the Variant Surface Glycoprotein (VSG); serial replacement of VSG (i.e. antigenic variation) is a means of immune evasion and results in chronic infection [10]. African trypanosome genomes contain large *VSG* gene families [11]–[12], but mono-allelic expression of a single gene is ensured because transcription is restricted to telomeric *VSG* expression sites (ES) [13]–[15]. Several other Expression Site-Associated Genes (*ESAG1-12*; [16]–[18]) are located in the ES and are co-transcribed with the active *VSG* [19]–[20]; all but *ESAG8* are predicted or known to be cell surface-expressed [21]. *T. cruzi* and *L. major* also possess multi-copy surface glycoprotein families (i.e. mucins and amastins respectively) but these are unrelated to VSG [8]–[9]. Indeed, *Leishmania* promastigotes have a largely non-proteinaceous, lipophosphoglycan-based surface coat [9].

Hence, while *T. brucei*, *T. cruzi* and *L. major* have physiological similarities associated with shared ancestry, the cell-surface architectures are highly divergent, reflecting the evolution of specific mechanisms for immune evasion and survival by each parasite [22]. A principal objective of comparative genomics is to identify taxon-specific features that may plausibly explain such phenotypic differences. Despite their similarities *T. brucei*, *T. cruzi* and *L. major* diverged long ago; so surface features that appear exclusive when their genomes are compared are not necessarily species-specific, or diagnostic of the diseases they cause. In particular, it remains to be determined if the *T. brucei*-specific surface features identified from these initial comparisons are truly species- or disease-specific, or general features of all African trypanosomes. Comparisons between more closely related species are essential to resolving this issue.

We recently reported the draft genome sequences for *T. congolense*, the closest known relative of *T. brucei*, and *T. vivax*, a more distantly related species, and described the evolution of *VSG* genes in African trypanosomes [12]–[23]. All species cause chronic animal trypanosomiasis characterized by recurrent parasitaemia and antigenic variation, but subtle differences are present in their pathology, life cycle and host range. For example, *T. vivax* can cause hyperacute hemorrhagic disease in cattle typically with much higher mortality than other species [24]. In the Tsetse, *T. brucei* and *T. congolense* infect the midgut but then migrate to the salivary glands and proboscis respectively prior to transmission to the vertebrate. In contrast, *T. vivax* avoids the insect midgut, a feature that seems to facilitate wholly mechanical transmission and its colonization of Tsetse-free areas [24]. Further, all three species infect a wide range of domestic animals but only *T. brucei* has evolved human infectivity, probably on at least two occasions in east (*T. b. rhodesiense*) and west Africa (*T. b. gambiense*) respectively [25].

Cell surface-expressed gene families encode abundant proteins at the forefront of host-parasite interactions [8]–[9], [22], [26]–[27]. The major surface protease (MSP, or gp63) has multiple isoforms, one of which (MSP-B) is responsible for cell-surface remodelling prior to transmission into the vector [28]–[29]. Papain-type cysteine peptidase B and C (also known as cathepsin-L and -B) are strongly associated with virulence phenotypes, degrading host proteins [30]–[31] and facilitating parasite transversal of the blood-brain barrier [32]. Other gene families encode diverse cell surface receptors, e.g. adenylate cyclases [33], and membrane transporters that are essential for normal cell physiology, e.g. transferrin receptors (TFR) [34]. Hence, the cell surface is an intuitive place to begin exploring species differences and here we present phylogenetic analyses of all gene families with predicted cell-surface roles in African trypanosomes. Although we do not include low-copy number features or non-protein cell-surface components, which may be equally important in function, our detailed analysis of the principal cell-surface gene families presents a global picture of evolutionary change on the trypanosome cell-surface.

## Methods

### Data sources

The African trypanosome cell surface phylome is a collection of phylogenies for gene families with predicted cell surface expression. The approach is summarized in Figure S1. Phylogenies were estimated from sequence data accessed through the GeneDB portal [35] and extracted from four genome sequences: *Trypanosoma brucei* TREU927 [11], *T. congolense* IL3000 and *T. vivax* Y486 [12] and, to provide an outgroup in phylogenetic comparisons, *T. cruzi* CL Brener [5]. Genome sequencing and annotation methods have been described previously [6], [12].

### Sequence clustering and cluster refinement

All *T. brucei* genes with cell surface motifs, (i.e. a predicted signal peptide, a predicted GPI anchor or a *trans*-membrane helix) were extracted from the *T. brucei* 927 genome sequence. Genes annotated as ‘unlikely’ or with fewer than 100 codons were removed. Homologs to each *T. brucei* ‘surface’ gene were identified among all *T. brucei*, *T. congolense*, *T. vivax* and *T. cruzi* predicted genes using wuBLAST [36]. Where at least four homologs occurred in at least one species, this constituted a ‘family’ amenable to phylogenetic analysis. Surface-expressed genes with fewer than four homologs are recorded as singleton, paired and triplet sequences in tables available from the CSP webpage. After removing genes already identified as homologous to *T. brucei* genes (i.e. widespread gene families), the BLAST exercise was repeated for *T. congolense* and *T. vivax* genes to identify cases absent in *T. brucei*. Signal peptides were predicted using SignalP [37], GPI anchors were predicted using Fraganchor [38] and *trans*-membrane helices were predicted using TMHMM [39]. 205 ‘surface expressed’ families were reduced to 79 by removing cases of poor alignment (i.e. sequences that could not be aligned by eye), of mis-annotation (i.e. non-coding sequence), of redundancy (i.e. technical duplicates arising from alleles in the *T. congolense* genome that were separately assembled), of genes with known expression in mitochondrial, lysosomal or other internal membranes, and by combining families with overlapping homology. Surface-expressed families may have been omitted because they possess signal peptides, GPI anchors, or *trans*-membrane helices that cannot be reliably recognized by current methods, or because their 5′ or 3′ regions are mis-specified. Equally, spurious recognition of these domains in hypothetical proteins (mostly *T. vivax* families) cannot be excluded. Each family is given a ‘Fam’ number (0–81) as described in Table S1; note that for historical reasons, there is no Fam48 or 68.

### Evidence for transcription

Given that most species-specific genes are putative and encode hypothetical proteins, evidence in support of their coding status was gathered from three sources: i) transcriptomic studies of *T. brucei* [40]; ii) Expressed Sequence Tags (EST) in multiple life stages of *T. congolense* [41]; and iii) partial RNAseq data for bloodstream form *T. vivax* [12] mapped against the *T. vivax* genome using SMALT [42].

### Multiple sequence alignment

Translated nucleotide sequences for each family were aligned in ClustalW [43]; all multiple alignments were then manually edited in BioEdit 7.1.3. [44]. In most cases, the amino acid sequence alignment was used in phylogenetic analysis to reduce homoplasy, but nucleotide sequences were examined in cases of low sequence divergence. The rates of synonymous (*k_s_*) and non-synonymous substitutions (*k_a_*) per site were calculated for each alignment using KaKs Calculator 2.0 [45] to estimate within-family sequence diversity.

### Phylogenetic analysis

Bayesian phylogenies were estimated using MrBayes v3.2.1 [46] under these settings: Nruns = 4, Ngen = 5000000, samplefreq = 500 and default prior distribution. Nucleotide and amino acid sequence alignments were analyzed using GTR+Γ and WAG+Γ models respectively. Maximum likelihood phylogenies were estimated using PHYML v3.0 [47] under an LG+Γ model [48] for amino acid sequences or a GTR+Γ model for nucleotide sequences. Node support was assessed using 100 non-parametric bootstrap replicates in addition to Bayesian posterior probabilities. Trees were rooted using *T. cruzi* sequences, or otherwise mid-point rooted. *VSG* phylogenies were estimated using alignments of selected, full-length sequences representative of global diversity under different conditions, as described previously [12].

### Phylogenetic reconciliation

The CSP contains phylogenies of gene families drawn from multiple species. We can infer historical gene duplications and losses from comparison of gene family phylogenies with the overlying species evolution [49]–[50]. For each gene family, a fully binary, rooted gene tree was integrated across the species tree (i.e. [*T. brucei, T. congolense*], *T. vivax*], *T. cruzi*]) using NOTUNG 2.6 [51]. A parameter *ρ*, was calculated from the ratio of speciation duplications (i.e. nodes supporting orthologs in daughter species) to unilateral duplications (i.e. nodes supporting in-paralogs in the same species), adjusted for gene family size. *ρ* reflects the degree of gene family turnover (combined incidence of gene gain and loss); high values of *ρ* indicate a phylogeny with minimal turnover, in which most lineages are represented by orthologs in all species. Low values indicate a phylogeny with high turnover, in which ancestral genes are frequently lost and replaced by novel duplicates, resulting in clades of species-specific in-paralogs and minimal orthology.

### Relative rate analysis

Significant differences in evolutionary rate between two lineages were examined using relative rates tests (RRTs; [52]). Nucleotide sequence alignments combining a given lineage, its sister taxon and an out-group (as described in Tables 1 and 2) were created and evaluated with MEGA v5.05 [53]. Where a test lineage consisted of multiple paralogous genes, the average rate difference between all comparisons is reported.

**Table 1:** Examples of significant substitution rate asymmetry inferred by relative rates tests.

Fam	Relative rates test:					n	χ^2^	p
	In-group 1		In-group 2		Out-group			
46	*T. vivax*-specific MSP-C genes	TvY486_0023730	*T. congolense* ortholog	TcIL3000.10.2050	TcCLB_505931.20	5	4.16	0.044
58	*T. brucei*-specific MFS transporter genes	Tb927.7.5950	*T. congolense* sister clade	TcIL3000.7.5000	Tb927.8.1650	8	7.11	0.045
61	*T. congolense*-specific nucleobase transporter genes	TcIL3000.0.59630	Conserved chr11 locus	TcIL3000.11.3580	Tb11.02.1105	6	107.79	0.00001
61	*T. brucei*-specific subtelomeric nucleotide transporter genes	Tb09.v4.0106	Conserved chr9 locus	Tb09.160.5480	TcIL3000.9.2500	4	9.59	0.022
67	*T. congolense*-specific cysteine peptidase C genes*	TcIL3000.0.48140	*T. brucei* sister clade	Tb927.6.560	TvY486_0600060	7	8.11	0.0054
72	*T. congolense* tandem gene copies of a hypothetical protein	TcIL3000.8.6610	Positional homologs in *T. brucei*	Tb927.8.6710	TvY486_0806350	9	9.85	0.0053
75	*T. congolense* tandem gene copies of a hypothetical protein	TcIL3000.0.05220	Positional homologs in *T. brucei*	Tb927.8.3880	TvY486_0803310	3	6.51	0.0012

Note: results are averaged across multiple comparisons of paralogous genes (n).

* Previously described [107] and divided into functionally distinct variants ‘CBs’ and ‘CBc’; this significant result relates only to ‘CBs’ genes. ‘CBc’ genes returned a non-significant result.

**Table 2:** Taxonomic distribution and sequence properties of *ESAG* gene families in African trypanosomes.

ESAG	n	Taxonomic distribution^a^:				Reciprocal monophyly^c^	Sequence diversity^d^:	Relative rates test^e^:						PHI statistic^f^:	
		Tb	Tco	Tv	Tc			Sites (bp)	ESAG	In-group	Out-group	χ^2^	p	ESAG	non-ES
1	21	+				yes	0.3832 (0.22)	1014	-	-	-	-	-	<0.0001	<0.0001
2	18	+	+^b^	∼		yes	0.1346 (0.11)	1308	211	TcIL3000.0.12020 (195)	TcIL3000.0.58770	0.63	0.427	<0.0001	<0.0001
3	112	+	∼	∼	∼	no	0.1758 (0.11)	882	176	Tb09.244.2060 (111)	TvY486_0042500	14.72	<0.001	<0.0001	<0.0001
4	53	+	∼	∼	∼	yes	0.1672 (0.34)	3429	259	Tb11.01.8820 (160)	TcIL3000.11.16970	23.39	<0.0001	<0.0001	<0.0001
5	8	+	∼	∼	∼	yes	0.0576 (0.02)	1237	252	Tb927.5.340 (212)	Tb927.4.810	6.32	0.012	<0.0001	0.267
6	1	+	+	∼		yes	0.0518 (0.02)	906	119	TcIL3000.0.50020 (131)	TcIL3000.0.03060	0.58	0.448	<0.0001	-
7	1	+	∼	∼		yes	0.0481 (0.01)	894	34	*ESAG6* (30)	TcIL3000.0.10990	0.25	0.617	<0.0001	-
8	2	+	∼	∼	∼	yes	0.0231 (0.01)	-	-	-	-	-	-	<0.0001	-
9	17	+			∼	yes	0.7639 (0.70)	972	-	-	-	-	-	<0.0001	0.777
10	7	+	∼	∼	∼	yes	0.0168 (0.01)	1812	44	Tb927.8.3620 (58)	TcIL3000.0.06950	1.92	0.165	1	0.106
11	12	+	∼	∼		no	0.1117 (0.05)	1134	-	-	-	-	-	0.127	<0.0001
12	1	+				yes	0.0998 (0.05)	300	-	-	-	-	-	0.142	-

Note: *ESAG* family size is given for non-ES linked (i.e. core or subtelomeric) copies.

a Plus and tilde symbols indicate the presence of orthologs and homologs respectively in *T. brucei* (Tb), *T. congolense* IL3000 (Tco), *T. vivax* Y486 (Tv) and *T. cruzi* CL Brener (Tc).

b While the closest relatives of *ESAG2* are in *T. congolense*, orthologs cannot be precisely defined among the *T. congolense VSG* repertoire.

c Reciprocal monophyly is confirmed where mutually exclusive clades of ES-linked and non-ES gene copies occur.

d Average pair-wise sequence divergence estimated from Bayesian phylogenies using RAxML 7.0.4. [108].

e Estimated using MEGA v5 [53] where appropriate out-groups are present; the number of unique differences per lineage given is given in brackets.

f Estimated using PhiPack [54] for separate alignments of *ESAGs* and related, non-ES gene copies (where non-ES copies are present).

### Recombination analysis

Phylogenetic incompatibility describes the presence of multiple phylogenetic signals within a single sequence alignment and is the historical signature of recombination. The Pair-wise Homoplasy Index (PHI) detects incompatibility between sites and is robust in the presence of rate heterogeneity [54], which might otherwise simulate the effects of recombination. P<0.05 for PHI indicates significant incompatibility between sites within an alignment, consistent with recombination. For each *ESAG* family, the index was calculated using PhiPack [54] for separate alignments of *ESAGs sensu stricto* and of homologous sequences from non-ES loci (unless these are largely absent, i.e. *ESAG6/7*, *8* and *12*).

### Analysis of gene expression

To determine mRNA expression levels for a single Fam50 family member (Tb927.7.380), quantitative real-time polymerase chain reaction (qRT-PCR) was carried out on total RNA extracted using RNeasy Mini Kit (QIAGEN). cDNA was generated using SuperScript II reverse transcriptase according to the manufacturer's instructions. qRT-PCR was carried out using three different isolated mRNA samples from four life-cycle stages (*in vitro* cultured bloodstream-stage and procyclic forms; *in vivo* cultured short stumpy bloodstream-stage; and *in vivo* cultured *T. brucei* bloodstream-stage). *T. brucei* Rab11 was used as a control to determine relative quantities of mRNA. The relative abundance of specific RNA was subsequently determined.

### Transfection and protein localization

Fam1 (i.e. Tb927.6.1310) and Fam50 (i.e. Tb927.7.380) genes were synthesized by Eurogentec. Tb927.6.1310 is the most divergent of all Fam1 gene copies, so it was selected for the benefit of targeting a single copy gene in localization experiments. Tb927.7.380 is also one of five tandem copies, and was selected because it was expressed to the greatest level for all paralogs in qPCR analyses. *T. brucei* single marker bloodstream line cells were cultured in HMI-9 medium as described previously [55]. Ectopic expression of haemagglutinin (HA) epitope-tagged Tb927.6.1310/Tb927.7.380 at the N-terminus (following the predicted signal peptide sequence) was carried out using pXS5/pDEX-577 [56] constitutive and inducible expression vectors respectively. For Western blotting, proteins were transferred onto Immobilon (polyvinyildene fluoride) membranes and incubated with primary mouse anti-HA antibody (1∶8,000) and subsequently with secondary rabbit anti-mouse peroxidase conjugate antibody (1∶10,000, Sigma). Immunofluorescence microscopy was carried out on permeabilised and non-permeabilised transfected cells harvested at log phase.

## Results

**Figure 1:**
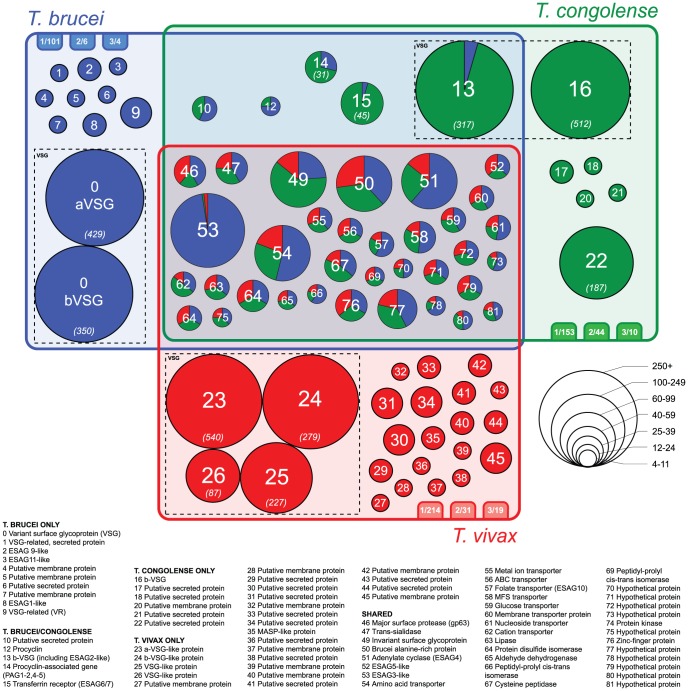
The taxonomic distribution of gene families in the cell-surface phylome, displayed in a Venn diagram. Phylogenies for all families are available through GeneDB. Each circle represents a family (i.e. >3 gene copies). The label in each circle refers to the description key, while size reflects the number of genes it contains; for large families the absolute number is shown in parentheses. For families present in multiple species, a pie chart is shown indicating relative gene numbers. The three tabs attending each species domain show the number of single-copy genes, pairs and triplets also predicted to have cell surface roles and to be species-specific (e.g. 101 singletons in *T. brucei*).

We estimated phylogenies using Maximum Likelihood and Bayesian methods for 79 gene families in African trypanosomes with known or predicted cell-surface location. This cell-surface phylome describes how these families have diversified during the evolution of African trypanosomes, and also identifies species-specific gene families, many of which remain uncharacterized. Taken together, the CSP shows that the cell-surface architecture evolved in the common ancestor of all African trypanosomatids, and has subsequently experienced subtle changes in individual lineages, suggesting the adaptation of their common inheritance. The CSP is described in a Venn diagram (Figure 1) and in Table S1. Throughout, gene families are referred to by their ‘Fam’ number (0–81; see methods). All sequence alignments, Hidden Markov Models (HMMs) and phylogenetic trees can be accessed at: http://www.genedb.org/Page/trypanosoma_surface_phylome.

### Phylogenetic diversity in conserved cell surface-expressed gene families

The conserved elements of the CSP, at the centre of Figure 1, generally contain cell-surface features that have been well described, including most known principal parasite effectors (i.e. MSPs, cathepsins and *trans*-sialidases) [26]–[27]. By contrast, genes at the periphery of Figure 1 are species-specific and mostly uncharacterized, even when they have given names in *T. brucei*; only 8/45 species-specific families (Fam0, 2, 3, 8, 12, 14–16) are characterized to some extent (e.g. by cellular localization) and function is only well known for two (*VSG* and *ESAG6*/7). Naturally, many trypanosome cell-surface proteins perform basic functions that are constrained by selection, resulting in small species differences (e.g. Fam54-56, 59-60, 62-65, 69-76 and 78-81). However, a widespread family is not necessarily unchanged, and the phylogenies of several conserved families involved in host-parasite interaction indicate surface proteome differences between species that could have functional implications.

In *T. vivax*, whole lineages have been lost, and on multiple occasions; for example among *trans*-sialidase genes (see Fam47 CSP page), there are no *T. vivax* orthologs to basal-branching lineages represented in *T. brucei* by Tb927.5.440 and Tb927.2.5280, which are otherwise widespread. Similarly, there are only three Major Facilitator Superfamily (MFS) transporters loci in *T. vivax* compared with six in *T. brucei* (see Fam58 CSP page), and no orthologs to the Proteins Associated with Differentiation (*PAD*) genes, one of which encodes a carboxylate transporter implicated in differentiation from vertebrate to insect life stages in *T. brucei* (i.e. Tb927.7.5930; [57]). Such within-family losses may coincide with the expansion of the remaining lineages. For instance *MSP-B* (Fam46) is present in *T. brucei*, *T. congolense* and the outgroup *T. cruzi*, but is absent from *T. vivax*; (a result confirmed by searching *T. vivax* unassembled reads for reciprocal BLASTx matches to *MSP-B*). This coincides with the evolution of 11 *MSP-C* genes in *T. vivax*, a gene that is single-copy in all other species (see Fam46 CSP page, and Table S1).

The surface functional repertoire also diverges through gene gain, for example among Fam61 genes (nucleoside/nucleobase transporters), required to scavenge host purines and are functionally differentiated with respect to both parasite life stage and substrate [58]–[61]. The Fam61 phylogeny shows that multiple gene duplications have occurred in both *T. brucei* and *T. congolense* (see Fam61 CSP page). However, while *T. brucei* has elaborated its *nucleoside* transporter lineage, producing four species-specific loci from a single-copy ancestral locus (probably Tb09.160.5480), *T. congolense* instead diversified its *nucleobase* transporter lineage, with 18 gene copies compared with three in *T. brucei* and five in *T. vivax*. This is not simply a difference in gene dosage, or an artifact of sequence assembly, since seven of these *T. congolense*-specific transporters (e.g. TcIL3000.0.12740) have a highly derived predicted protein sequence, lacking ∼130 amino acids from the 3′ end and displaying only 39% amino acid identity with the *T. congolense* chromosome 11 isoform (54% similarity), and which itself displays 54% identity and 66% similarity with its *T. brucei* ortholog. Therefore, these genes are predicted to encode proteins with signal peptides and eight *trans*-membrane helices, but lack the canonical C-terminus of the conserved nucleobase transporter including its GPI-anchor signal.

The combined effect of gene gains and losses, i.e. gene family turnover, is reflected in the topology of phylogenies. Typically, gene families predate contemporary genomes, and orthologs in each species of each ancestral gene form a clade in the phylogeny. Examples of this familiar pattern in trypanosomes naturally include structural or metabolic gene families displaying little innovation [62]–[64], as well as some CSP families including Fam56 (ABC transporters) and Fam65 (aldehyde dehydrogenase), although the majority of these genes are likely intracellular. Many cell surface-expressed gene families similarly originate prior to contemporary species, but their tree topologies indicate greater post-speciation innovation. To investigate the extent to which species derive novel genes post-speciation, we calculated *ρ* for each family, the ratio of orthology (DIV) to paralogy (DUP), corrected for gene family size, and where DIV is the incidence of gene divergence through speciation and DUP is the incidence of gene duplication, inferred through phylogenetic reconciliation (Table S1). Families like Fam56 (*ρ* = 0.67) and Fam65 (*ρ* = 0.73) possess high *ρ* values, indicating that most loci are retained in all species; for example, across 22 ABC transporter loci there are no unilateral gene duplications and only 7 gene losses (2 in *T. brucei/T. congolense*, 1 in *T. congolense* and 4 in *T. vivax*). While these losses probably have functionally consequence, Fam56 and similar examples have a relatively constant gene complement.

Conversely, many familiar cell surface components have *ρ*<0.05, indicating that gene copies cluster more by species than by locus, i.e. recent paralogy rather than ancient orthology. Fam54 (amino acid transporters; *ρ* = 0.006), Fam58 (MFS transporters; *ρ* = 0.018) and Fam61 (nucleoside transporters; *ρ* = 0.01) all display low *ρ* values due to *T. brucei*-specific expansions (see individual CSP pages), which occurs against a general background of conservation. This cannot be said for phylogenies for other families, e.g. Fam46 (*ρ* = 0.013), Fam49 (*ρ* = 0.002), Fam50 (*ρ* = 0.003), Fam67 (*ρ* = 0.003), and Fam77 (*ρ* = 0.002), which consist of species-specific clades of highly similar, tandem duplicates, at one or a few conserved loci. Fam47 (*trans*-sialidase; *ρ* = 0.025) and Fam51 (adenylate cyclase; *ρ* = 0.002) provide examples intermediate between the first and second patterns, with *T. brucei* and *T. congolense* possessing orthologs to conserved loci, while all *T. vivax* genes are monophyletic and hence lack orthology with other species.

Gene family diversification is a product of both gene duplication and sequence divergence [65], so even where gene repertoire is constant, significant asymmetry in nucleotide substitution rates between ancestral and duplicated lineages may indicate that important functional change has occurred in either lineage. Previously, we have identified frequent rate asymmetry following gene duplication of amino acid transporters (Fam54) in *T. brucei* [66]. Further examples are evident in the CSP. For instance, branch lengths among cysteine peptidase B (Fam67) genes in *T. congolense* (average genetic distance = 0.092, n = 16) are significantly longer than in *T. brucei* (0.0037, n = 11, p<0.0001; t-test) or *T. vivax* (0.016, n = 6, p<0.0001). *T. congolense* cysteine peptidase B includes structural variants with distinct catalytic functionality [67], which is clearly absent from *T. brucei*. Table 1 records this and other cases of rate asymmetry involving species-specific expansions, further details of which are provided in each CSP family page.

### The transferrin receptor (*TFR*) gene family evolved in the ancestor of *T. brucei* and *T. congolense*

A TFR is expressed in bloodstream form *T. brucei* and is required for iron uptake [68]. It is not homologous with its mammalian counterpart, and they function quite differently [68]. The trypanosome TFR is a GPI-anchored heterodimer encoded by paralogous gene families *ESAG6* and *7* (Fam15; [68]–[70]). *ESAG7* is 57 amino acids shorter than *ESAG6* and encodes a protein without a GPI-anchor signal, but otherwise the genes are very closely related [71]. When present, *ESAG6* and *7* are found in tandem immediately downstream of the ES promoter. Outside of the ES, genes homologous to *ESAG6/7* in *T. brucei* 927 consist of a single *ESAG6/7* tandem pair (Tb927.7.3250/3260) at a strand-switch region on chromosome 7, probably representing a secondary transposition from an ES, and the Procyclin-Associated Genes (*PAG1*, *2*, *4* and *5*; Fam14), which are adjacent to the procyclin loci [72]. *ESAG6* and *7* and the *PAGs* are homologous to the a-type *VSGs* (a-*VSG*; [69], [71]), leading to the suggestion that the TFR derives from VSG [26], [73].

**Figure 2:**
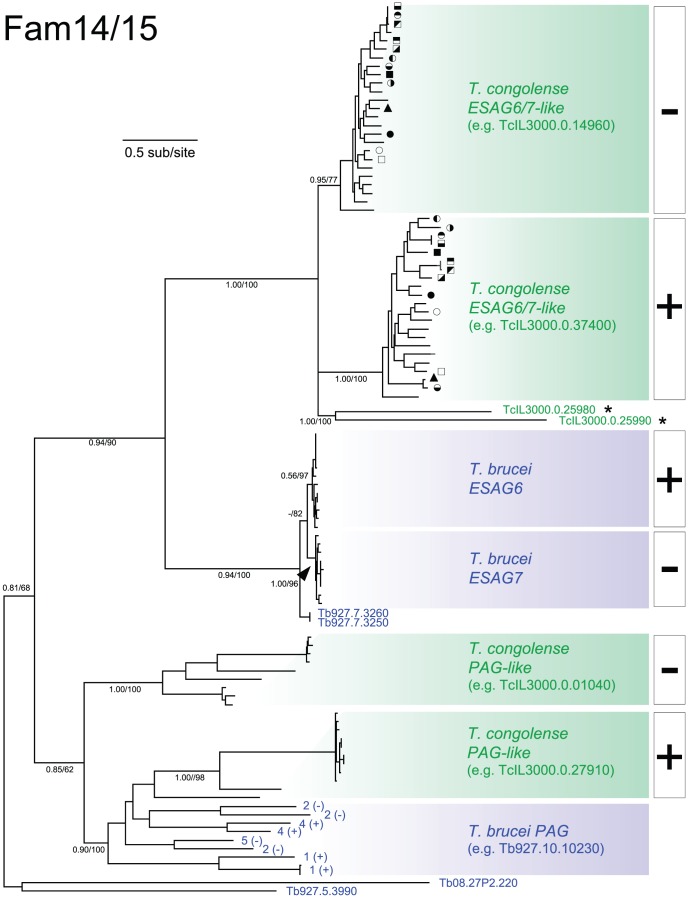
Bayesian phylogeny of transferrin receptor-like genes in *T. brucei* and *T. congolense* (Fam14/15). The phylogeny was estimated from an amino acid sequence alignment of 342 characters including all *ESAG6*-like proteins from *T. brucei* 927 (light blue) and *T. congolense* IL3000 (green), as well as *ESAG6/7* sensu stricto from *T. brucei* 927, 427 and *T. b. gambiense* 972 (dark blue). A mixed amino acid substitution strategy was applied with default settings using MrBayes v3.2.1. The phylogeny is rooted using an outgroup of two a-type VSG protein sequences from *T. brucei*. Bayesian posterior probability/non-parametric bootstrap values are provided for selected nodes. Terminal nodes that describe sequences derived from tandem pairs in *T. congolense* are labeled with common symbols. Terminal nodes representing Procyclin-Associated Genes (*PAG1*, *2*, *4* and *5*) are numbered. Throughout, the presence or absence of predicted GPI anchor signals is noted using + and − respectively.

The *T. congolense* genome contains 45 genes (in Fam15) that are homologous to *ESAG6/7*, plus 31 genes (in Fam14) whose closest sequence match is to *PAGs* in *T. brucei*. We refer to both Fam14 and Fam15 as *TFR*-like genes. Figure 2 describes the phylogeny for *TFR*-like genes and shows that the *T. congolense* genes are paraphyletic, that is, there are two clades (Fam14 and 15) each more closely related to sequences in *T. brucei* (*PAG* and *ESAG6/7* respectively) than to each other. Given the homology between a-*VSG* and *TFR* genes, this shows that Fam14 and 15 are not a-*VSG* (of which *T. congolense* has none; [12]) because they are much closer to *T. brucei TFR* than a-*VSG*. The *T. vivax* genome contains a-*VSG*-like genes but these have an equally distant relationship to both *ESAG6/7* and a-*VSG* in *T. brucei*, and significantly are not part of the *TFR* gene family of *T. brucei* and *T. congolense* [12]. Therefore, genes that now encode TFR proteins, and others associated with procyclin expression sites in *T. brucei*, likely evolved before the speciation of *T. brucei* and *T. congolense*, but after the separation from *T. vivax*.

The essential difference between *TFR* genes in *T. brucei* and *T. congolense* is genomic distribution. While *ESAG6/7* are almost exclusively found in ESs, *T. congolense* orthologs are distributed widely among subtelomeres and not usually close to telomeres. Nevertheless, phylogenetic and sequence comparisons suggest that *TFR* function is conserved in *T. congolense*. First, like *ESAG6/7*, Fam15 genes in *T. congolense* split into two equal-sized sister clades, encoding proteins that differ in the prediction of a GPI anchor (Figure 2). Second, just as *ESAG6* and *7* are typically arranged in GPI+/GPI− tandem pairs in *T. brucei*, 28/45 of *T. congolense* genes are also arranged in tandem pairs at subtelomeric loci, each pair combining representatives from the GPI+ and GPI− clades. Finally, amino acid positions within the transferrin binding domain [71] are conserved in all *ESAG6/7*, *PAG* and their *T. congolense* orthologs (see Fam15 CSP page). These results suggest that an orthologous TFR is present in *T. brucei and T. congolense* but not *T. vivax*.

### An expanded insect stage-specific surface glycoprotein gene family conserved across African trypanosomes

**Figure 3:**
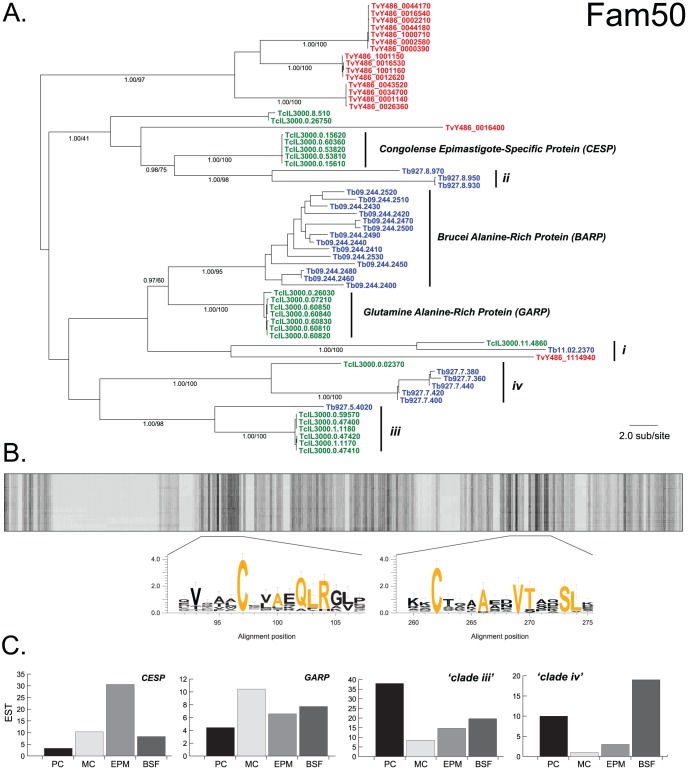
Bayesian phylogeny and expression of *BARP*/*GARP*-like genes (Fam50). **A**. The phylogram was estimated from a multiple protein sequence alignment of 307 characters. The tree is mid-point rooted. Selected nodes are supported by posterior probability values and non-parametric bootstraps generated from a maximum likelihood analysis using an LG model with rate heterogeneity. **B**. Cartoon of sequence conservation across the Fam50 protein sequence alignment, darker shading reflects conservation. Two conserved regions are expanded to show sequence motifs in WebLogo v2.8.2. format [106]; ubiquitous residues are shaded red. **C**. Histograms showing the number of *T. congolense* EST corresponding to each of four clades in A, (*CESP*, *GARP*, subfamily ‘*iii*’ and subfamily ‘*iv*’), recovered from four life stages: procyclic (PC), metacyclic (MC), epimastigote (EPM) and bloodstream form (BSF); data from [41].

In addition to procyclin and VSG, *T. brucei* and *T. congolense* possess a third, highly abundant major surface glycoprotein expressed during the insect stage. These are known as *Brucei* Alanine-Rich Protein (BARP; [74]) and Glutamine Alanine-Rich Protein (GARP; [75]–[78]) respectively. Although GARP was initially thought analogous to procyclin in *T. brucei* [76], a procyclin ortholog was subsequently identified in *T. congolense* [78] and the CSP confirms a widespread procyclin family (Fam12). Structural affinities between *GARP*, which is expressed most strongly in epimastigotes [79], and *BARP*, which is epimastigote-specific [74], have been demonstrated [80], and the CSP confirms these two gene families as sister taxa (see Figure 3A). *T. vivax* contains 15 genes encoding BARP/GARP-like proteins that form three distinct subfamilies; each subfamily encodes proteins with distinctive repetitive domains towards the N-terminus that are absent in other species. Unfortunately, poor assembly in these regions prevents us from discerning their genomic organization, but at least some are arranged in tandem as in *T. brucei* and *T. congolense*.

*BARP*, *GARP* and their *T. vivax* homologs are part of a larger gene family (Fam50) in the CSP. We identified conserved sequence regions that unite these familiar families with other insect stage-specific genes and several hypothetical or uncharacterized genes (Figure 3B). The region at positions 262–274 contains a ubiquitous cysteine residue, followed four positions downstream by a VTxxSL motif in *BARP* and *GARP*, which is present with slight variations in all family members. A single-copy locus on chromosome 11 (i.e. Tb11.02.2370/TcIL3000.11.4860; marked ‘*i*’) is the sister clade to *BARP/GARP* and may be present in *T. vivax* also (TvY486_11149440). The *Congolense* Epimastigote-Specific Protein (*CESP*) gene family is expressed in *T. congolense* epimastigotes only, where it may have a role in adhesion to host surfaces [81]. Figure 3A shows that *CESP* has a sister clade in *T. brucei* comprising a tandem gene array on chromosome 8 (marked ‘*ii*); notably, these genes may be preferentially expressed in insect salivary glands [82], i.e. the location of *T. brucei* epimastigotes. An ortholog to *CESP* may be present in *T. vivax* (i.e. TvY486_0016400), although the position of this gene is not robust.

In addition to *GARP* and *CESP*, the CSP identified two related subfamilies encoding hypothetical proteins, (marked ‘*iii*’ and ‘*iv*’), which comprise subtelomeric tandem gene arrays. Analysis of stage-defined *T. congolense* EST (Figure 3C; [41]) proteomic analysis [83] found that subfamily ‘*iii*’ is preferentially expressed in *T. congolense* procyclic stage (see Figure 3C). Accordingly, the single-copy ortholog to subfamily ‘*iii*’ in *T. brucei* (Tb927.5.4020) is also preferentially expressed in procyclic cells based on transcriptome data [40], [84]; and a recent qRT-PCR analysis identified transcripts corresponding to Tb927.5.4020 in the insect midgut, although protein expression was not examined [82]. Subfamily ‘*iv*’ comprises sequences on chromosome 7 in *T. brucei* (i.e. Tb927.7.360) and a single-copy ortholog in *T. congolense* (i.e. TcIL3000.0.02370). In transcriptomic studies of *T. brucei*, expression data for these genes was weak and inconclusive [40]. However, qRT-PCR in various insect tissues suggests significant up-regulation of Tb927.7.360 (and paralogs) in the insect salivary gland and in metacyclic trypomastigotes [82]. Quantitative proteomic analysis in *T. congolense* indicated 13-fold higher expression of TcIL3000.0.02370 in epimastigotes over procyclics [83]. Hence, it seems likely that subfamily ‘*iv*’ genes are expressed during the insect-to-vertebrate transition.

**Figure 4:**
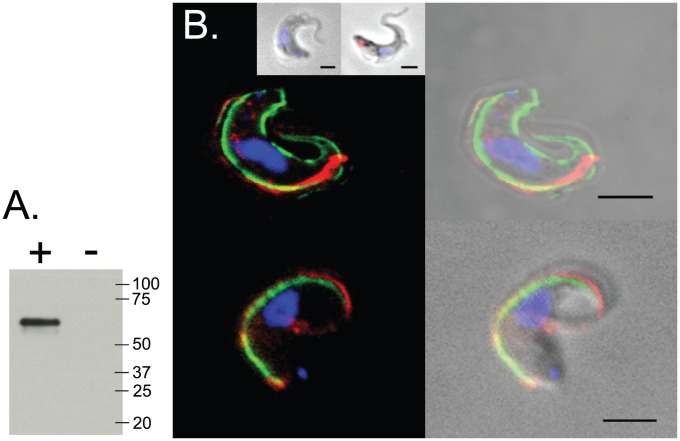
Expression of a Fam50 gene (Tb927.7.380) in *T. brucei*. **A**. Western blot; 2×10^7^ cells were sampled from either induced (+) or uninduced (−) cells after 1-day induction with 1 µg/mL tetracycline. Markers shown in kDa. **B**. Immunofluorescence analysis of Tb927.7.380 expression in *T. brucei*, showing co-staining of non-permeabilised cells with plasma membrane protein VSG 221 (*top*) and PFR2 (*bottom*). *Left*: Merged images with color combination for DAPI-stain of the nucleus and kinetoplast (blue), fluorescent stain of HA epitope tag (red) and VSG/PFR fluorescence (green). *Right*: merged pictures from phase and fluorescence. *Inset*: co-staining of non-permeabilised and permeabilised cells with Rab11 (intracellular marker). No fluorescence was seen from non-transfected cells (data not shown). GFP expressed from pDEX-577 vector localised to the cytoplasm (data not shown). Scale bar is 2 µm.

To localize expression of a single gene copy of subfamily ‘*iv*’ in *T. brucei*, Tb927.7.380 was haemagglutinin (HA) epitope-tagged at the N-terminus (following the predicted signal peptide sequence) and expressed ectopically using a pDEX-577 inducible-expression vector. Protein expression was confirmed by Western blot (Figure 4A), and immuno-fluorescence microscopy indicates that Tb927.7.380 protein co-localizes with paraflagellar rod protein 2, consistent with specific expression at, or close to, the flagellar membrane (Figure 4B).

### Expression site-associated gene families (*ESAGs*) evolved uniquely in *T. brucei*

*ESAGs* have homologs outside of *T. brucei* [85]–[86], but these may only represent distant relationships within widely conserved protein families. With complete genome sequences for *T. congolense* and *T. vivax* we can now examine evidence for true *orthology* and therefore, the possibility that *ESAG* phylogenetic lineages predate *T. brucei* (Table 2). Orthologous lineages of *TFR* genes (i.e. *ESAG6/7*) are present in *T. congolense* and we have previously argued that *ESAG2* belongs to a widespread lineage most closely related to b-type *VSG* in *T. congolense* [12]. Altogether, we find evidence that the *T. congolense* and *T. vivax* genomes contain homologous sequences to 9 of 12 *ESAG* families, while *ESAG9* may have homologs in *T. cruzi* [87] (Table 2). Two trends emerge from phylogenies for each *ESAG* family shown in their individual CSP pages. First, *ESAGs* from multiple *T. brucei* strains are monophyletic and therefore, have a single origin; and second, with the exception of *ESAG6/7*, the sister clades to *ESAGs* are not orthologs in other species but chromosomal-internal genes in *T. brucei*. We interpret this as evidence for origins post-speciation, i.e. *ESAGs* are *T. brucei*-specific. Examining these closest relatives outside of the expression sites provides some indication of the origins of *ESAGs*, as demonstrated by Fam51, i.e. *ESAG4* and the adenylate cyclases.

**Figure 5:**
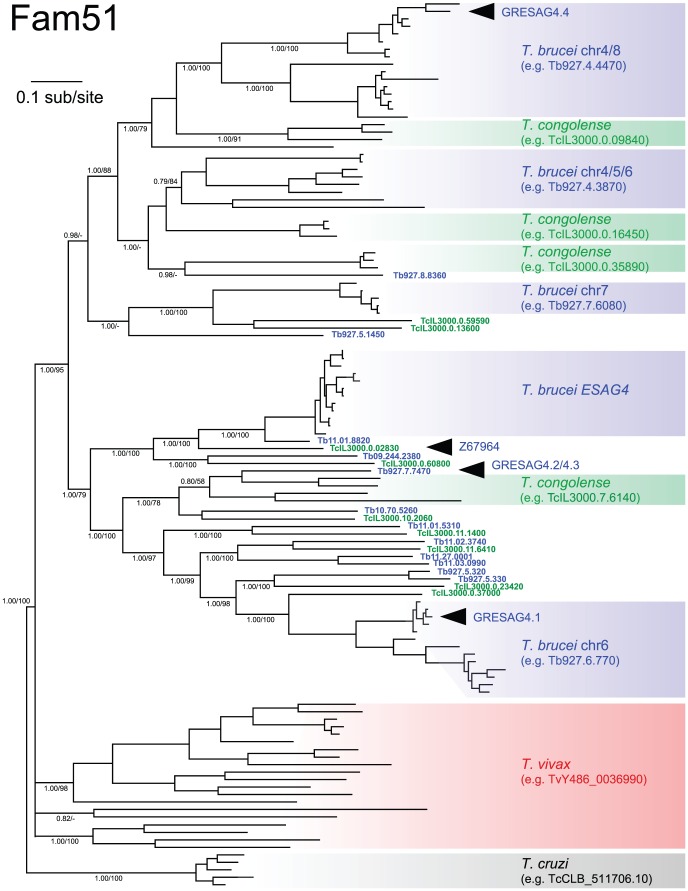
Bayesian phylogeny of adenylate cyclase genes from African trypanosomes (Fam51). The phylogeny was estimated from an amino acid sequence alignment of 1239 characters including all adenylate cyclase proteins from *T. brucei* 927, *T. congolense* IL3000 and *T. vivax* Y486, as well as *ESAG4 sensu stricto* from *T. brucei* 927, 427 and *T. b. gambiense* 972. A mixed amino acid substitution strategy was applied with default settings using MrBayes v3.2.1. The phylogeny is rooted using an outgroup of selected *T. cruzi* homologs that represent total diversity. Bayesian posterior probability/non-parametric bootstrap values are provided for selected nodes. Black arrows denote the positions of previously named ‘*GRESAG4*’ sequences, as well as an *ESAG4*-like cDNA from *T. congolense* (Z67964; [85]).

*Trans*-membrane adenylate cyclases are conserved across Trypanosomatids [88]–[89], and comprise a large gene family with diverse roles in *T. brucei* [85], [90]–[91]. *ESAG4* is one lineage expressed specifically in the bloodstream stage, and instrumental in inhibiting host innate immunity [91]. The *T. congolense* and *T. vivax* genome sequences include 34 and 24 adenylate cyclase genes respectively. The adenylate cyclase phylogeny (Figure 5) shows that *T. brucei* and *T. congolense* lineages are paraphyletic, and in 10 cases *T. brucei* genes have orthologs in *T. congolense* that are positionally conserved. However, there are no orthologs of *ESAG4* among *T. congolense* homologs. Indeed, the most closely related gene to *ESAG4* is Tb11.01.8820, located at the subtelomeric boundary of chromosome 11. This gene has an ortholog in *T. congolense* (TcIL3000.11.16970), which is syntenic. Relative rates tests show that the substitution rate of *ESAG4* has accelerated significantly compared with Tb11.01.8820 (p<0.0001; Table 2). Comparison of Tb11.01.8820 and *ESAG4* sequences (Figure S2) shows that this remodelling has primarily affected the intracellular domains. 245 amino acid differences are distributed preferentially towards the C-terminal, with 69% occurring after the putative *trans*-membrane helix (a portion accounting for only 35% of total characters). Furthermore, of 54 sites where Tb11.01.8820 and TcIL3000.11.16970 are conserved, but *ESAG4* is derived (i.e. unambiguous *ESAG4* apomorphies), 41 occur in the intracellular domain. While the adenylate cyclase catalytic domain is intracellular, the evolution of *ESAG4* has not altered the 8 residues identified as important for catalytic function [92]. Hence, *ESAG4* represents a *T. brucei*-specific expansion of adenylate cyclase genes, most likely initiated through the transposition of a conserved locus to the ES, and coinciding with derivation of the protein structures associated with signal transduction within the cell but not catalysis.

The detail presented for other *ESAGs* in their CSP pages suggests that, like *ESAG4* and Tb11.01.8820, *ESAGs* themselves are *T. brucei*-specific but descended from conserved genes, typically members of multi-copy families with subtelomeric distributions in several species. For example, *ESAG2* and *ESAG6/7* were, as previously noted, derived from *VSG* [12], [68]. *ESAG3*- and *ESAG5*-like loci are on *T. vivax* contigs containing telomeric repeats (GenBank accessions HE578915 and HE578917), but not *VSG*. *ESAG8*, although not surface-expressed, is most closely related to two leucine-rich repeat protein (*LRRP*) genes (i.e. Tb927.1.3670 and Tb927.3.580), that are chromosome-internal and include nuclear localization signal and RING motifs, which are diagnostic of *ESAG8* [93]. While these two genes are *T. brucei*-specific, they are more closely related to conserved *LRRP* genes, suggesting that they may be progenitors of *ESAG8*. Finally, on the Fam3 CSP page, a structural comparison of *ESAG11* and Invariant Surface Glycoprotein (*ISG*) sequences indicates that ESAG11 is homologous to ISG and so perhaps a highly modified derivative of these widespread surface proteins [94]. As Table 2 shows, only *ESAG1* and *ESAG12* appear to have no homology beyond *T. brucei*, suggesting that they have evolved *de novo* within the ES.

### Species-specific genes include a family derived from b-type *VSG* and expressed in the flagellar pocket of *T. brucei*

Besides *ESAGs*, the CSP contains various species-specific genes (Table S1). *T. brucei*-specific gene families include Fam4-7 encoding hypothetical proteins with predicted signal peptides but no similarity to known proteins. Fam4-7 genes are all adjacent to strand-switch regions and typically arrayed in tandem; transcriptomic studies suggest that they are expressed preferentially or solely in bloodstream forms [40], [84], [95]. The *VSG*-related (*VR*) genes previously identified in *T. brucei* [96] are also specific to *T. brucei*, although similar in structure to canonical *VSG* in *T. congolense* [12]. Finally, the CSP contains another family of *VSG*-like genes unique to *T. brucei*: Fam1.

**Figure 6:**
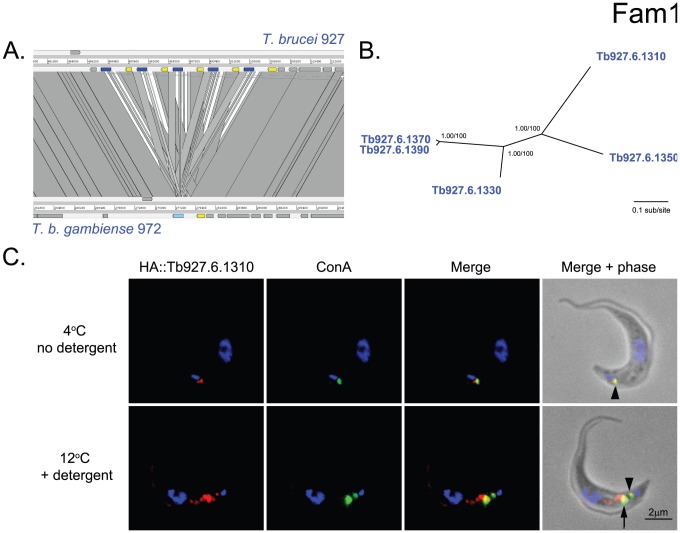
Phylogeny and expression of a *T. brucei*-specific, VSG-like hypothetical protein (Fam1). **A**. Fam1 consists of five, non-identical tandem gene copies at a strand-switch region on chromosome 5, which is unique to *T. brucei*. **B**. A Bayesian phylogram estimated from a multiple nucleotide sequence alignment of 1068 characters. The tree is midpoint-rooted. Nodes are supported by posterior probability values and non-parametric bootstraps generated from a maximum likelihood analysis using a GTR+G model. **C**. Immunofluorescence analysis of Tb927.6.1310 expression in *T. brucei*. Tb927.6.1310 was N-terminally HA epitope-tagged and expressed in bloodstream-form cells. Cells expressing HA::Tb927.6.1310 were loaded with FITC-concanavalin-A in serum-free media and incubated at either 4°C (upper panel) or 12°C (lower panel). ConA is restricted to the flagellar pocket at the lower temperature, whereas it is transported to, and trapped within, Rab5A positive early endosomes at 12°C. Columns in each panel (from left to right); fluorescent stain of HA epitope tag (red); FITC-ConA fluorescence (green); merged images for fluorescence; merged images from phase and fluorescence. DAPI-stain of the nucleus and kinetoplast is shown in blue. HA::Tb927.6.1310 co-localizes with ConA at the flagellar pocket (indicated with arrow head) and early endosomes (indicated with a whole arrow).

In *T. brucei* 927, Fam1 comprises a polymorphic tandem array of 5 copies (0.1–3.2% nucleotide sequence divergence) at a strand-switch region on chromosome 6 (Figure 6A). Comparison with *T. b. gambiense* 972 indicates that Fam1 copy number may differ between strains because only a single gene (corresponding to the divergent 5′-most copy, Tb927.6.1310, Figure 6B) is present. The gene encodes a 347 amino acid protein with a predicted signal peptide and GPI-anchor. Fam1 genes are homologous to b-type *VSG*, but lack the typical C-terminal domain of canonical *VSGs* [12]. qRT-PCR analysis indicated that Tb927.6.1310 is predominantly expressed in bloodstream stages [12]. Enrichment of Tb927.6.1310 transcripts has been observed in metacyclic forms in the insect salivary gland [82], but this remains to be verified at the protein level.

We expressed the gene product of Tb927.6.1310 using a constitutive expression system (pXS5), and tagged at the N-terminus of the mature protein with an HA-9 epitope (Figure 6C). The HA epitope was placed two residues downstream of the predicted N-terminus of the mature protein following signal sequence processing. By Western analysis a single band was detected migrating at ∼45 kDa in whole cell lysates. However, the predicted molecular weight of the protein is ∼39 kDa, suggesting glycosylation at either or both predicted N-glycosylation sites. Cells were stained with a monoclonal antibody against HA and counterstained with FITC-concanavalin A. At 4°C, the fusion protein clearly colocalized with conA, conditions which block endocytosis and so retain the lectin exclusively within the flagellar pocket, a subdomain of the plasma membrane, and therefore demonstrating access to the cell surface. When cells were permeabilised with detergent, it was clear that Tb927.6.1310 protein was also present in additional internal compartments, and based on partial overlap of conA at 12°C (which retains conA in the flagellar pocket and early endosomes) and HA signals, these structures likely correspond to early and/or recycling endosomes. Hence, we conclude that the Tb927.6.1310 gene product is present at the parasite surface and may be restricted to the flagellar pocket, which is frequently observed for low abundance GPI-anchored proteins in this organism, and Tb927.6.1310 is also present within the endosomal apparatus.

In *T. congolense*, Fam22 is the most abundant species-specific gene family with >100 copies. Fam22 genes are distributed throughout putative subtelomeric regions and are typically situated immediately downstream of *VSG*. *T. congolense* VSG 3′UTR's are too short, (often only 15–30 bp; [41]) for Fam22 to fall within these regions. qRT-PCR analysis identified Fam22 sequences in all life stages except bloodstream forms (J. Donelson, unpublished data), but it is unclear whether Fam22 is a novel family of coding sequences or a non-coding, regulatory sequence. Nevertheless, Fam22 sequences are highly abundant. *Trypanosoma vivax* has substantially more species-specific gene families (19; Fam27-45) than either other species, which may be expected given that *T. vivax* is the natural outgroup to *T. brucei* and *T. congolense*. None have any significant similarity with known protein structures and more transcriptomic and proteomic surveys will be required to confirm that these sequence families genuinely encode *T. vivax*-specific proteins. However, many of these putative gene families are abundant (e.g. Fam31 and Fam34 have 38 and 34 members respectively) and transcripts corresponding to several gene families are among bloodstream-form RNA-seq data (Fam29-32, 34-35, 38-39; see Table S1).

## Discussion

The ancestor of *T. brucei*, *T. congolense* and *T. vivax* was very likely a hemoparasite of vertebrates, spread by Tsetse flies, and likewise fully exposed to the host immune response during its period in the mammalian host. Most familiar cell-surface features – both physiological regulators such as membrane transporters and disease effectors such as MSP and cathepsin – were already present in the ancestor. This is intuitive given that these features are typically present in *T. cruzi*. However, the CSP shows that the peculiar nature of the *T. brucei* cell surface, dominated by *VSG* [12], *BARP/GARP*-like genes and procyclin (Fam12) during various life-stages, also appears to have originated in the ancestral African trypanosome.

The role of the TFR on the ancestral cell-surface is more debatable. *ESAG6/7* are thought to have evolved from a-*VSG* variant antigens [26], [73] but we show that the sister clade to *ESAG6/7* are *T. congolense* Fam15 genes, which do not encode any known variant antigens [12]. Rather than originating from a-*VSG* in *T. brucei*, phylogenetic analysis of all *VSG*-like sequences [see Fam0 CSP pages] indicates that *TFR*-like sequences evolved from an a-*VSG*-like gene, (and further differentiated into *ESAG6*- and *PAG*-like genes), in the *T. brucei/T. congolense* ancestor, after separation from the lineage leading to *T. vivax*. While there are no *TFR*-like sequences in *T. vivax*, this does not preclude an analogous transferrin receptor in this species, since there is a large and structurally diverse a-*VSG*-like family (Fam23 [12]), the functional diversity of which is unknown. In short, we predict that Fam15 genes in *T. congolense* also encode a heterodimeric transferrin receptor, orthologous to the *T. brucei* TFR.

However, if the *T. brucei/T. congolense* ancestor possessed an orthologous heterodimeric TFR comprising GPI+ and GPI− monomers, we would expect GPI+ genes from *T. brucei* and *T. congolense* to be sister taxa reflecting their ancestry, and likewise for GPI−. Yet a literal interpretation of Figure 2 suggests separate expansions of Fam15 genes in each species, and thus independent origins of GPI+/− isoforms. Furthermore, branches separating *ESAG6* and *7* (average genetic distance (p) = 0.114, n = 21) are much shorter than distances among the *T. congolense* genes (p = 0.604, n = 49), implying a recent origin for *ESAG7* from *ESAG6* through the deletion of its C-terminus. We consider this to reflect rapid turnover post-speciation of *TFR*-like genes that evolved in the ancestor, rather than independent origins, which is less parsimonious. Indeed, the same pattern of reciprocal monophyly between species is seen in other phylogenies (e.g. *VSG*, Fam50, Fam67), but it is clearly unparsimonious to suggest recent origins for these widely conserved families. Gene turnover replaces ancestral-type genes with more derived types post-speciation resulting in concerted evolution, a process exacerbated by recombination among tandem gene duplicates [97], and causing any signature of orthology to be ‘overwritten’ [98]. Such processes are known to affect *ESAG6/7* routinely [20], [99] and frequent transposition of Fam15 genes between *T. congolense* subtelomeres is also apparent (data not shown). Given that this molecular evolution introduces phylogenetic artefacts, the Fam15 phylogeny need not refute the most parsimonious hypothesis that a TFR protein originated in the *T. brucei/T. congolense* ancestor.

While the essential character of the cell surface was established in the ancestral trypanosome, this common inheritance has been adapted subsequently. The evolution of *ESAGs* in *T. brucei*, uniquely linked to the telomeric *VSG* expression site, is a principal example of species-specific genomic adaptation. In some cases we can identify the likely origin of *ESAG* lineages among chromosome-internal loci; *ESAGs* 3, 4, 5 and 10 are derived from conserved loci that can be located precisely [85]–[86], [100]. *ESAGs* 2 and 6/7 are derived from variant antigen genes that evolved in the *T. brucei*/*T. congolense* ancestor [12]. *ESAGs* 8, 9 and 11 have more remote homology to conserved subtelomeric gene families, i.e. *LRRP* [101], *MASP* [87] and *ISG* (see Fam3 CSP page) respectively. This suggests a scenario in which genes with existing subtelomeric distributions (except *ESAG10*) and cell-surface roles (except *ESAG8*) were progressively compartmentalized into an independently-promoted telomeric locus, perhaps to provide a more precise regulatory environment.

Like the origin of Fam1 in *T. brucei*, the evolution of the ES demonstrates how novel cell-surface genes are repeatedly derived from existing major surface glycoproteins, whose abundance seems to provide a reservoir of raw material for neofunctionalization. Although *ESAG* functions are obscure, *ESAG* phylogenies suggest that they are distinct from those of conserved genes from which *ESAGs* evolved and indispensable on an evolutionary timescale. *ESAGs* from different *T. brucei* strains are monophyletic (except *ESAG3*), indicating no frequent transposition of sequences between ES and non-ES loci. *ESAG*-related genes at chromosome-internal loci are not observed in the ES and do not recombine with *ESAGs*, despite very frequent recombination among ES and non-ES copies respectively [20], [99], [102]. So although previous work has reported that *ESAGs* are not essential in the short term [101]–[102], the association between *ESAG* sequences *sensu stricto* and the telomeric ES has been preserved by selection over the long term, suggesting that *ESAG* and *ESAG*-like functions are distinct and non-redundant.

The CSP emphasizes dramatic cases of gene gain such as *ESAGs* in *T. brucei*, but significant phenotypic differences, such as life cycle variation, could be due to relatively subtle differences in conserved gene families such as Fam50. Given that *BARP*, *GARP* and *CESP* are preferentially expressed in the epimastigote stage [74], [79], [81] and that transcriptome data for both *T. congolense* and *T. brucei* indicate that subfamilies ‘*iii*’ and ‘*iv*’ are associated with insect mid-gut and salivary gland stages respectively [82], we suggest that Fam50 ranks alongside procyclin and VSG as a major surface glycoprotein, specifically related to the insect-to-vertebrate transition in multiple species. This is especially interesting because of the developmental variation among African trypanosomes during this transition. Unlike *T. brucei* and *T. congolense*, *T. vivax* remains within the insect mouthparts after feeding; this could reflect the basal-branching position of *T. vivax* in the species phylogeny (i.e. *T. vivax* is plesiomorphic and never evolved a mid-gut stage) or secondary loss (i.e. a mid-gut stage is the ancestral state). *T. vivax* also has a relatively small Fam50 repertoire, lacking orthologs to three clades: *BARP/GARP* and subfamilies ‘*iii*’ and ‘*iv*’. These genes might have evolved in the *T. brucei/T. congolense* ancestor if *T. vivax* is plesiomorphic, in which case all *T. vivax* genes should branch towards the root. Yet two of five Fam50 lineages in *T. vivax*, (i.e. TvY486_0016400 and TvY486_1114940), are nested among the would-be *T. brucei/T. congolense* gains. Reconciliation of this topology with the species tree indicates that if functionality is absent in *T. vivax*, this is due to secondary loss, rather than *T. brucei/T. congolense* gain.

Having systematically analyzed protein coding sequences for species differences, it is particularly important to remember that the cell-surface architecture comprises much more than the proteins encoded by the genes in the CSP and that non-proteinaceous elements, not least the surrounding glycocalyx composed of the carbohydrate moieties attached to membrane glycoproteins and glycolipids, might be equally important in determining phenotypic variation. Experimental studies of the cell-surface demonstrate that non-protein glycoconjugates could play an equal role in regulating host-parasite interactions, for example, a protease-resistant surface molecule (PRS) is known to dominate the surface of procyclic-stage *T. conglolense* [79]. *T. brucei* expresses various glycoconjugates on their surfaces that only become apparent in null mutants that cannot express the major surface glycoprotein [103]–[104]. Even considering the protein component, low abundance genes not considered in the CSP may still perform a vital role; for example, the haptoglobin-hemoglobin receptor (Tb927.6.440; [105]) responsible for resistance to trypanolytic factor by *T. brucei* is single-copy.

### Conclusion

The essential character of genes expressed on African trypanosomes cell-surfaces was largely established in the common ancestor. Subsequently, prominent families have experienced rapid turnover of phylogenetic diversity, indicating both functional dynamism and redundancy. As we distinguish the functions of family members, we should be mindful of where orthology is absent and where it is retained; the latter, for example among *MSP* subtypes, cathepsin-L and B, or *ESAG6*-like and *PAG*-like *TFR* genes, is a strong indication of long-term functional differentiation and non-redundancy among paralogs. Truly species-specific genes represent adaptations of this shared inheritance and, in *T. brucei*, include almost all *ESAGs* as well as various GPI-anchored glycoproteins associated with strand-switch regions (Fam4-7). We anticipate that with improved genome assembly, species-specific genes, perhaps analogous to *ESAGs*, will be revealed in *T. congolense* and *T. vivax* also. To this extent, comparative genomics has met its objectives and the challenge now is to define how these unique genes and variants influence phenotypic differences in biology and disease.

## Supporting Information

Figure S1 Flowchart describing how the cell-surface phylome was compiled.
(EPS)

Figure S2 Distribution of unambiguous, apomorphic characters in ESAG4. The figure shows an amino acid sequence alignment for four ESAG4 proteins and Tb11.01.8820, the most related non-ES homolog (at top). Identical residues are represented with a dot. Positions conserved in ESAG4 only are shaded red. The location of the predicted *trans*-membrane helix (green) and adenyly cyclase catalytic Pfam domain (yellow) are marked on the Tb11.01.8820 sequence. ESAG4 apomorphies, i.e. characters that have changed in ESAG4 but remained constant in Tb11.01.8820 and its ortholog in *T. congolense* (TcIL3000.11.16970), are marked with an asterisk.
(EPS)

Table S1 Gene families comprising the cell surface phylome.
(DOCX)
