# Supplementary material for: A Cell-surface Phylome for African Trypanosomes
Source: PLoS Negl Trop Dis. 2013 Mar 21;7(3):e2121. doi: 10.1371/journal.pntd.0002121 (PMC3605285; doi:10.1371/journal.pntd.0002121)
Supplement: Table S1 — Gene families comprising the cell surface phylome. (DOCX) [file pntd.0002121.s003.docx]

**Table S1**. Gene families comprising the cell surface phylome.

|  |  |  |  |  |  |  |  |  |  |  |  |  |  |  |  |  |  |
| --- | --- | --- | --- | --- | --- | --- | --- | --- | --- | --- | --- | --- | --- | --- | --- | --- | --- |
| **Family** | **Example ID** | **Product description** | **Structure^a^** | |  | **Taxa^b^** | |  |  | **Evidence of transcription^c^** | | |  |  |  |  |  |
|  |  |  | SP | GPI | TMH | *Tbb* | *Tco* | *Tv* | TOTAL | cBF | SL-BF | ST-BF | PF-log | PF-stat |  |  |  |
|  |  |  |  |  |  |  |  |  |  |  |  |  |  |  |  |  |  |
| ***T. brucei*-specific:** | |  |  |  |  |  |  |  |  |  |  |  |  |  |  |  |  |
|  |  |  |  |  |  |  |  |  |  |  |  |  |  |  |  |  |  |
| 0a | Tb11.0640 | variant surface glycoprotein (a-type) | y | y | 0 | 459 | 0 | 0 | 459 | 14604 | 12866 | 2542 | 197 | 319 |  |  |  |
| 0b | Tb10.v4.0031 | variant surface glycoprotein (b-type) | y | y | 0 | 380 | 0 | 0 | 380 | 25767 | 16671 | 1244 | 167 | 181 |  |  |  |
| 1 | Tb927.6.1390 | hypothetical protein | y | y | 0 | 5 | 0 | 0 | 5 | 15058 | 15164 | 13803 | 6380 | 9653 |  |  |  |
| 2 | Tb09.160.5400 | hypothetical protein (ESAG9) | y | y | 0 | 15 | 0 | 0 | 15 | 4589 | 6579 | 43669 | 645 | 379 |  |  |  |
| 3 | Tb927.4.3270 | ESAG11-related protein | y | y | 0 | 7 | 0 | 0 | 7 | 1538 | 1492 | 615 | 1473 | 365 |  |  |  |
| 4 | Tb927.4.3260 | hypothetical protein | y | n | 1 | 4 | 0 | 0 | 4 | 915 | 679 | 457 | 837 | 265 |  |  |  |
| 5 | Tb927.3.600 | hypothetical protein | y | y | 1 | 8 | 0 | 0 | 8 | 13864 | 14725 | 14113 | 3602 | 2381 |  |  |  |
| 6 | Tb927.3.5700 | hypothetical protein | y | y | 0 | 5 | 0 | 0 | 5 | 6280 | 8311 | 9500 | 2404 | 582 |  |  |  |
| 7 | Tb09.v1.0540 | hypothetical protein | y | n | 1 | 10 | 0 | 0 | 10 | 3111 | 3400 | 3918 | 1322 | 962 |  |  |  |
| 8 | Tb09.142.0310 | ESAG1 | y | n | 0 | 17 | 0 | 0 | 17 | 2969 | 5176 | 2844 | 550 | 760 |  |  |  |
| 9 | Tb927.1.5060 | variant surface glycoprotein-related | y | y | 0 | 35 | 0 | 0 | 35 | 5925 | 6625 | 8507 | 2380 | 2618 |  |  |  |
|  |  |  |  |  |  |  |  |  |  |  |  |  |  |  |  |  |  |
| ***T. brucei*- *T. congolense*** | |  |  |  |  |  |  |  |  |  |  |  |  |  |  |  |  |
|  |  |  |  |  |  |  |  |  |  |  |  |  |  |  |  |  |  |
| 10 | Tb927.7.6600 | hypothetical protein | y | n | 0 | 8 | 6 | 0 | 14 | 9375 | 4949 | 6068 | 2498 | 3276 |  |  |  |
| 12 | Tb927.6.510 | GPEET2 procyclin precursor | y | y | 1 | 6 | 2 | 0 | 8 | 1768 | 1014 | 7966 | 52410 | 46689 |  |  |  |
| 13 | Tb927.6.470 | ESAG2 | y | y | 0 | 12 | 254 | 0 | 266 | 7380 | 5291 | 7144 | 6554 | 7652 |  |  |  |
| 14 | Tb11.01.6220 | procyclin-associated gene 4 (PAG4) protein | y | y | 0 | 9 | 22 | 0 | 31 | 10444 | 12196 | 9204 | 2476 | 894 |  |  |  |
| 15 | Tb927.7.3250 | transferrin receptor (ESAG6/7) | y | y | 0 | 2 | 43 | 0 | 45 | 6263 | 8251 | 9526 | 1987 | 1592 |  |  |  |
|  |  |  |  |  |  |  |  |  |  |  |  |  |  |  |  |  |  |
| ***T. congolense*-specific:** | |  |  |  |  |  |  |  |  | **Evidence of transcription^d^** | | |  |  |  |  |  |
|  |  |  |  |  |  |  |  |  |  | PCF | MCF | EMF | BSF | n |  |  |  |
|  |  |  |  |  |  |  |  |  |  |  |  |  |  |  |  |  |  |
| 16 | TcIL3000.0.18060 | variant surface glycoprotein | y | y | 0 | 0 | 338 | 0 | 338 | 0 | 4 | 4 | 13 | 21 |  | | |
| 17 | TcIL3000.0.20120 | hypothetical protein | y | n | 0 | 0 | 20 | 0 | 20 | 0 | 0 | 0 | 0 | 0 |  |  |  |
| 18 | TcIL3000.0.00720 | hypothetical protein | y | n | 0 | 0 | 12 | 0 | 12 | 1 | 0 | 1 | 0 | 2 |  | | |
| 20 | TcIL3000.0.06070 | hypothetical protein | y | n | 1 | 0 | 5 | 0 | 5 | 0 | 2 | 1 | 0 | 3 |  | | |
| 21 | TcIL3000.0.06530 | hypothetical protein | y | n | 0 | 0 | 9 | 0 | 9 | 2 | 1 | 1 | 0 | 4 |  | | |
| 22 | TcIL3000.0.01360 | VSG-associated hypothetical protein | y | n | 2 | 0 | 187 | 0 | 187 | 0 | 1 | 0 | 0 | 1 |  | | |
|  |  |  |  |  |  |  |  |  |  |  |  |  |  |  |  |  |  |
| ***T. vivax*-specific:** | |  |  |  |  |  |  |  |  | **Evidence of transcription^e^** | | |  |  |  |  |  |
|  |  |  |  |  |  |  |  |  |  |  |  |  |  |  |  |  |  |
|  |  |  |  |  |  |  |  |  |  |  |  |  |  |  |  |  |  |
| 23 | TvY486_0001620 | variant surface glycoprotein | y | n | 0 | 0 | 0 | 478 | 478 | TvY486_0900440 (99%) | | |  |  |  |  |  |
| 24 | TvY486_0043480 | variant surface glycoprotein-like | y | y | 0 | 0 | 0 | 211 | 211 | TvY486_0028795 (99%) | | |  |  |  |  |  |
| 25 | TvY486_0005290 | variant surface glycoprotein-like | y | y | 0 | 0 | 0 | 133 | 133 | TvY486_0026030 (94%) | | |  |  |  |  |  |
| 26 | TvY486_0016660 | variant surface glycoprotein-like | y | y | 0 | 0 | 0 | 64 | 64 | TvY486_0002910 (93%) | | |  |  |  |  |  |
| 27 | TvY486_0043530 | hypothetical protein | y | y | 1 | 0 | 0 | 5 | 5 | - |  |  |  |  |  |  |  |
| 28 | TvY486_0001150 | hypothetical protein | y | n | 1 | 0 | 0 | 6 | 6 | - |  |  |  |  |  |  |  |
| 29 | TvY486_0001600 | hypothetical protein | y | n | 0 | 0 | 0 | 21 | 21 | TvY486_0035060 (21%) | | |  |  |  |  |  |
| 30 | TvY486_0001730 | hypothetical protein | y | n | 0 | 0 | 0 | 29 | 29 | TvY486_0025925 (46%) | | |  |  |  |  |  |
| 31 | TvY486_0002090 | hypothetical protein | y | n | 0 | 0 | 0 | 38 | 38 | TvY486_0000210 (26%) | | |  |  |  |  |  |
| 32 | TvY486_0002120 | hypothetical protein | y | n | 1 | 0 | 0 | 8 | 8 | TvY486_0004160 (59%) | | |  |  |  |  |  |
| 33 | TvY486_0003230 | hypothetical protein | y | y | 0 | 0 | 0 | 14 | 14 | - |  |  |  |  |  |  |  |
| 34 | TvY486_0004790 | hypothetical protein | y | y | 0 | 0 | 0 | 34 | 34 | TvY486_0024150 (90%) | | |  |  |  |  |  |
| 35 | TvY486_0004880 | TcMUCII-like hypothetical protein | y | y | 0 | 0 | 0 | 18 | 18 | TvY486_0044810 (20%) | | |  |  |  |  |  |
| 36 | TvY486_0004900 | hypothetical protein | y | y | 0 | 0 | 0 | 5 | 5 | - |  |  |  |  |  |  |  |
| 37 | TvY486_0010370 | hypothetical protein | y | n | 1 | 0 | 0 | 5 | 5 | - |  |  |  |  |  |  |  |
| 38 | TvY486_0012580 | hypothetical protein | y | y | 0 | 0 | 0 | 5 | 5 | TvY486_0045330 (36%) | | |  |  |  |  |  |
| 39 | TvY486_0014250 | hypothetical protein | y | n | 1 | 0 | 0 | 7 | 7 | TvY486_0014250 (62%) | | |  |  |  |  |  |
| 40 | TvY486_0015690 | hypothetical protein | y | n | 1 | 0 | 0 | 13 | 13 | - |  |  |  |  |  |  |  |
| 41 | TvY486_0017190 | hypothetical protein | y | n | 0 | 0 | 0 | 19 | 19 | - |  |  |  |  |  |  |  |
| 42 | TvY486_0019770 | hypothetical protein | y | n | 1 | 0 | 0 | 13 | 13 | - |  |  |  |  |  |  |  |
| 43 | TvY486_0021360 | hypothetical protein | y | y | 0 | 0 | 0 | 8 | 8 | - |  |  |  |  |  |  |  |
| 44 | TvY486_0000190 | hypothetical protein | y | n | 0 | 0 | 0 | 8 | 8 | - |  |  |  |  |  |  |  |
| 45 | TvY486_0003000 | hypothetical protein | y | n | 1 | 0 | 0 | 16 | 16 | - |  |  |  |  |  |  |  |
|  |  |  |  |  |  |  |  |  |  |  |  |  |  |  |  |  |  |
|  |  |  |  |  |  |  |  |  |  | **Genetic divergence^f^:** | | |  | **Reconciliation analysis^g^:** | | | *ρ* |
| **Conserved:** | |  |  |  |  |  |  |  |  | *k_s_* |  | *k_a_* |  | DIV | DUP | LOSS |  |
|  |  |  |  |  |  |  |  |  |  |  |  |  |  |  |  |  |  |
| 46 | Tb927.8.1640 | major surface protease (gp63) | y | y | 1 | 13 | 6 | 12 | 31 | 3.08 | 0.29 | 0.30 | 0.07 | 10 | 25 | 1 | 0.013 |
| 47 | Tb927.7.6850 | trans-sialidase | y | y | 0 | 10 | 8 | 6 | 24 | 2.76 | 0.25 | 0.35 | 0.04 | 10 | 17 | 3 | 0.025 |
| 49 | Tb927.5.620 | invariant surface glycoprotein | y | n | 1 | 19 | 49 | 11 | 79 | 1.43 | 0.18 | 0.86 | 0.07 | 10 | 71 | 3 | 0.002 |
| 50 | Tb09.244.2400 | brucei alanine-rich protein | y | n | 0 | 24 | 22 | 17 | 63 | 1.56 | 0.19 | 0.77 | 0.08 | 9 | 53 | 2 | 0.003 |
| 51 | Tb10.70.5260 | adenylate cyclase | y | n | 1 | 53 | 21 | 12 | 86 | 2.72 | 0.22 | 0.37 | 0.07 | 15 | 81 | 2 | 0.002 |
| 52 | TcIL3000.0.09680 | ESAG5-like | y | n | 1 | 7 | 5 | 7 | 19 | 2.14 | 0.34 | 0.61 | 0.11 | 5 | 14 | 1 | 0.019 |
| 53 | Tb927.2.2020 | ESAG3 | y | n | 2 | 123 | 1 | 2 | 126 | 2.11 | 0.00 | 0.69 | 0.00 | 1 | 122 | 0 | 0.001 |
| 54 | Tb927.8.7610 | amino acid transporter | n | n | 13 | 40 | 20 | 14 | 74 | 3.02 | 0.41 | 0.16 | 0.06 | 24 | 57 | 3 | 0.006 |
| 55 | Tb927.8.7560 | metal ion transporter | n | n | 1 | 5 | 4 | 4 | 13 | 3.00 | 0.40 | 0.24 | 0.08 | 7 | 7 | 0 | 0.077 |
| 56 | Tb927.8.2380 | ABC transporter | y | n | 9 | 21 | 21 | 18 | 60 | 2.43 | 0.56 | 0.14 | 0.10 | 40 | 1 | 9 | 0.667 |
| 57 | Tb927.8.3620 | folate/biopterin transporter | n | n | 12 | 7 | 3 | 2 | 12 | 3.07 | 0.56 | 0.20 | 0.07 | 4 | 9 | 1 | 0.037 |
| 58 | Tb927.8.1650 | MFS transporter | y | n | 13 | 16 | 10 | 5 | 31 | 3.04 | 0.37 | 0.20 | 0.02 | 12 | 21 | 3 | 0.018 |
| 59 | Tb927.4.2290 | glucose transporter | n | n | 11 | 5 | 4 | 3 | 12 | 2.95 | 0.26 | 0.24 | 0.06 | 6 | 3 | 0 | 0.167 |
| 60 | Tb927.5.2430 | membrane transporter protein | y | n | 11 | 5 | 3 | 4 | 12 | 3.01 | 0.37 | 0.16 | 0.04 | 8 | 2 | 1 | 0.333 |
| 61 | Tb927.6.220 | purine nucleoside transporter | y | n | 10 | 9 | 4 | 4 | 17 | 3.09 | 0.15 | 0.24 | 0.02 | 6 | 35 | 0 | 0.010 |
| 62 | Tb11.01.0720 | cation transporter | n | n | 8 | 5 | 7 | 2 | 14 | 3.05 | 0.14 | 0.21 | 0.02 | 4 | 9 | 0 | 0.032 |
| 63 | Tb927.8.7440 | lipase | n | n | 1 | 5 | 5 | 4 | 14 | 2.66 | 0.47 | 0.34 | 0.20 | 8 | 4 | 2 | 0.143 |
| 64 | Tb927.7.5790 | protein disulfide isomerase | y | n | 0 | 9 | 9 | 9 | 27 | 2.25 | 0.79 | 0.17 | 0.09 | 22 | 1 | 5 | 0.815 |
| 65 | Tb927.6.4210 | aldehyde dehydrogenase | y | n | 0 | 4 | 4 | 3 | 11 | 2.40 | 0.21 | 0.12 | 0.06 | 8 | 1 | 1 | 0.727 |
| 66 | Tb927.8.2000 | cyclophilin-type isomerase | y | n | 1 | 14 | 15 | 18 | 47 | 2.73 | 0.77 | 0.12 | 0.08 | 26 | 7 | 1 | 0.079 |
| 67 | Tb927.6.560 | cysteine peptidase (cathepsin) | y | n | 0 | 12 | 15 | 6 | 33 | 3.24 | 0.62 | 0.21 | 0.01 | 4 | 43 | 0 | 0.003 |
| 69 | Tb10.61.0760 | FKBP-type isomerase | y | n | 0 | 5 | 5 | 5 | 15 | 2.72 | 0.63 | 0.19 | 0.18 | 10 | 0.01 | 0 | 0.667 |
| 70 | Tb927.8.7720 | hypothetical protein | y | n | 1 | 4 | 2 | 2 | 8 | 2.22 | 0.18 | 0.57 | 0.04 | 2 | 8 | 0 | 0.031 |
| 71 | Tb927.8.1850 | hypothetical protein | y | n | 0 | 7 | 7 | 6 | 20 | 3.39 | 2.08 | 0.40 | 0.50 | 8 | 8 | 0 | 0.050 |
| 72 | Tb927.8.6730 | hypothetical protein | y | n | 6 | 5 | 5 | 4 | 14 | 2.42 | 0.56 | 0.53 | 0.19 | 4 | 10 | 0 | 0.029 |
| 73 | Tb927.8.4100 | hypothetical protein | n | n | 2 | 4 | 2 | 1 | 7 | 2.52 | 0.08 | 0.45 | 0.06 | 2 | 3 | 0 | 0.095 |
| 74 | Tb927.8.4000 | hypothetical protein (ABC1 protein kinase) | y | n | 2 | 5 | 5 | 5 | 15 | 3.33 | 0.23 | 0.18 | 0.07 | 10 | 0.01 | 0 | 0.667 |
| 75 | Tb927.8.3900 | hypothetical protein (extensin signature) | y | n | 2 | 3 | 3 | 2 | 8 | 3.70 | 0.41 | 0.29 | 0.06 | 2 | 9 | 0 | 0.028 |
| 76 | Tb927.8.3400 | hypothetical protein (zinc-finger protein) | y | n | 4 | 12 | 11 | 13 | 36 | 3.18 | 0.73 | 0.22 | 0.08 | 24 | 1 | 4 | 0.667 |
| 77 | Tb927.6.380 | hypothetical protein (C-lectin) | y | y | 2 | 20 | 16 | 5 | 41 | 2.65 | 0.16 | 0.53 | 0.05 | 3 | 30 | 0 | 0.002 |
| 78 | Tb927.7.6170 | hypothetical protein | n | n | 4 | 4 | 1 | 1 | 6 | 3.10 | 0.24 | 0.26 | 0.01 | 2 | 4 | 0 | 0.083 |
| 79 | Tb927.7.4280 | hypothetical protein | y | n | 2 | 4 | 4 | 4 | 12 | 1.90 | 0.48 | 0.70 | 0.18 | 4 | 7 | 1 | 0.048 |
| 80 | Tb927.4.5070 | hypothetical protein | n | n | 2 | 4 | 3 | 1 | 8 | 2.33 | 1.46 | 0.54 | 0.40 | 6 | 2 | 3 | 0.375 |
| 81 | Tb10.70.0040 | hypothetical protein | n | n | 12 | 4 | 3 | 2 | 9 | 3.01 | 0.12 | 0.23 | 0.05 | 2 | 7 | 0 | 0.032 |
|  |  |  |  |  |  |  |  |  |  |  |  |  |  |  |  |  |  |

^a^ Genes were selected for the surface phylome due to the presence of predicted signal peptide (SP), GPI-anchor (GPI) and/or *trans*-membrane helix (TMH) in their products.

^b^ The number of full-length, non-pseudogenic homologous sequences in each species. in the case of VSG, only full-length gene copies are counted.

^c^ Expression levels for each of four *T. brucei* life stages are given for each example ID: cultured bloodstream forms (cBF), slender bloodstream (SL-BF), stumpy bloodstream (ST-BL), logarithmic phase procyclic (log-PF) and stationary phase procyclic (stat-PF). Data from [40].

^d^ For each example ID, the number of expressed sequence tags (EST) recovered in total (n) and from each of four *T. congolense* life stage-specific libraries [41] is given. Life stages: procyclic (PCF), metacyclic (MCF), epimastigote (EMF), bloodstream-form (BSF).

^e^ Where detected among *T. vivax* bloodstream form RNAseq data [12], the most abundant gene family member is shown, with the percentage sequence coverage given in parentheses.

^f^ Average *k_a_* and *k_s_* calculated for *T. brucei - T.congolense* ortholog pairs inferred from Bayesian phylogenies using KaKs Calculator 2.0 [45]; each average is followed by one standard deviation.

^g^ Gene family trees were reconciled with species relationships with NOTUNG, using *T. cruzi* homologs as an outgroup, to quantify gene divergence due to speciation (DIV), in-species gene duplication (DUP), gene loss (LOSS) and the DIV:DUP ratio corrected for gene family size (*ρ*).
